# The Family Psychoeducation Fidelity Scale: Psychometric Properties

**DOI:** 10.1007/s10488-020-01040-3

**Published:** 2020-04-23

**Authors:** I. Joa, J. O. Johannessen, K. S. Heiervang, A. A. Sviland, H. A. Nordin, M. Landers, T. Ruud, R. E. Drake, G. R. Bond

**Affiliations:** 1 TIPS – Network for Clinical Research in Psychosis, Stavanger University Hospital, Post box 8100, 4068 Stavanger, Norway; 2 Network for Medical Sciences, Faculty of Health, University of Stavanger, Stavanger, Norway; 3 Division of Mental Health Services, Akershus University Hospital, Nordbyhagen, Norway; 4 Westat, USA; 5 Institute for Clinical Medicine, University of Oslo, Oslo, Norway

**Keywords:** FPE fidelity scale, Psychometric properties, Family psychoeducation, Psychosis

## Abstract

This study examined psychometric properties and feasibility of the *Family Psychoeducation (FPE) Fidelity Scale*. Fidelity assessors conducted reviews using the FPE fidelity scale four times over 18 months at five sites in Norway. After completing fidelity reviews, assessors rated feasibility of the fidelity review process. The FPE fidelity scale showed excellent interrater reliability (.99), interrater item agreement (88%), and internal consistency (mean = .84 across four time points). By the 18-month follow-up, all five sites increased fidelity and three reached adequate fidelity. Fidelity assessors rated feasibility as excellent. The FPE fidelity scale has good psychometric properties and is feasible for evaluating the implementation of FPE programs. *Trial registration* ClinicalTrials.gov Identifier: NCT03271242.

**Electronic supplementary material:**

The online version of this article (10.1007/s10488-020-01040-3) contains supplementary material, which is available to authorized users.

## Background

About one-third of the total population experiences a mental disorder during their lifetime. Therefore, a large proportion of the population have family members with mental health problems. Families have an important role in the caring for the ill individual (Awad and Voruganti 2008). The development of evidence-based interventions to support family involvement for people with severe mental illness has been a central feature of community-based mental health (Lobban et al. 2013; McWilliams et al. 2010; Yesufu-Udechuku et al. 2015). Rigorous research has demonstrated the value of involving the family early in treatment (Cabral and Chaves 2010; Day and Petrakis 2017; Jeppesen et al. 2005; McFarlane 2016; McWilliams et al. 2010; Nilsen et al. 2016; Nilsen et al. 2014; Pharoah et al. 2010). A Cochrane review concluded that family psychoeducation (FPE) reduced relapses and readmissions over a 12-month period for the clients with a psychotic disorder (Pharoah et al. 2010). The U.S. National Implementing Evidence-Based Practices Project included FPE as one of five core practices for routine mental health settings (Drake et al. 2001; McHugo et al. 2007).

FPE comprises a number of overlapping intervention models to provide families with education, skills training, and support. Mental health programs provide FPE in either single-family (Miklowitz et al. 2010) or multi-family (five to six families) formats (McFarlane et al. 2003) (McFarlane 2004). The multi-family FPE format used in the current study (the training included both models) included a number of meetings with patients and family members together, workshops for family members, workshops for patients, and a fortnightly multi-family group often extending over 2 years (Nielsen et al. (2014, 2016).

Despite strong evidence for the benefits of FPE, and that they are frequently applied in early intervention programs these programs are less implemented in more long term treatment settings (Eassom et al. 2014; Selick et al. 2017). Barriers include both the skills and the attitudes of the workforce as well as organizational and economic issues (Kavanagh et al. 1993). Facilitators include involving service users and advocacy groups in staff training, team-based training, ongoing clinical supervision, and commitment at the organizational and commissioning levels (Berry and Haddock 2008).

Evidence-based interventions require faithful implementation because programs that adhere to standards produce better outcomes (Bond et al. 2011; McHugo et al. 1999). Fidelity scales have therefore become useful tools for monitoring the implementation, enhancing both training of clinicians and quality of services (Lu et al. 2012). Fidelity monitoring is important for clinical research (Bond and Drake 2019) because without fidelity measurement it is not possible to distinguish failure of the intervention from failure to implement the intervention (Mowbray et al. 2003).

The *Family Psychoeducation (FPE) Fidelity Scale*, developed to measure implementation in the U.S. National Implementing Evidence-Based Practices Project (McHugo et al. 2007), established that FPE, with technical assistance, could be implemented to good fidelity within one year. The FPE fidelity scale is based on the core principles described by Dixon et al. (Dixon et al. 2001). It was initially designed to measure multi-family therapy (McFarlane et al. 2002) but the scale is flexible enough to be used for other family psychoeducation approach sharing the same principles and methods, including single family psychoeducation (Miklowitz et al. 2010).

The current study aimed to investigate the psychometric properties and clinical use of the FPE Fidelity Scale, including item analysis, interrater reliability, interrater item agreement, internal consistency, sensitivity to change, and feasibility.

## Methods

Data for the current analysis came from a Norwegian cluster-randomized trial on the implementation of evidence-based treatments for patients with psychosis. Five sites from the participating health trusts in Norway agreed after random assignment to implement the FPE treatment and to receive intensive technical assistance and implementation support from an expert. This sub-study assessed use of the FPE scale in these five sites. Informed consent: Informed consent was obtained from all individual participants included in the study.

### Study Sites

Five clinical sites, randomly assigned to implement FPE with supports, represented health trusts in urban and rural areas in Norway. Three of the sites were community mental health centers, one was a combined inpatient and outpatient site for assessment of persons with first—episode psychosis, and one was a child and adolescent outpatient clinic. Only one site (the first episode psychosis site) was using the structured FPE format at baseline.

### Procedures

Prior to initiating implementation of FPE, the research team provided to staff from all the study sites (experimental and control) a four-day workshop to introduce both the multi- and single-family FPE format. Each site sent at least two clinicians to the workshop. The research team developed an online toolkit and distributed it to all sites. This toolkit included a description of the evidence-based practice (key points from Norwegian guideline regarding FPE evidence) (Helsedirektoratet 2013), translated version of single and multifamily groups FPE manuals (McFarlane 2004; Miklowitz et al. 2010) and presentations from the workshop. The sites were offered clinical supervision focusing on cases by an FPE clinical expert weekly (first 6 months) to monthly (last 6 months) during the first year, all in a group format by video and telephone). In addition, the five experimental study sites was offered (on site) supervision on implementation and quality improvement (focus on FPE fidelity scale content) by a separate trained expert in implementation supervision every 2 week for 6 months and then monthly for 12 months.

The fidelity assessors were not involved as staff or in supervision in any FPE activity at the sites where they completed an assessment. The 15 fidelity assessors (who included psychiatrists, psychologists, mental health nurses and others with experiences as researchers and/or clinicians in treatment of psychosis) had been trained in doing the fidelity assessment of FPE. They subsequently completed ratings at each site at baseline and after 6, 12 and 18 months. To enhance reliability, two assessors completed each review, conducting a daylong site visit to gather information from the sources specified in the fidelity rating manual (including interview and reviewing written program material). During the site visit, the two assessors independently rated the items on the FPE fidelity scale. After completing their independent ratings, the fidelity assessors compared ratings, identified items on which their ratings disagreed, and reached a consensus rating through discussion. The fidelity raters participated in joint workshops that were held after each round of assessment discussing experiences and results for the fidelity assessment.

### Measures

The 14-item FPE Fidelity Scale (Dixon et al. 2001; McHugo et al. 2007) rates current behavior and organizational activities on 5-point, behaviorally-anchored items, ranging from 1 = not implemented to 5 = fully implemented, with a rating of 4.0 defined as adequate fidelity. For this study, we dropped the item on prodromal signs because prodromal patients were not included, using the remaining 13 items as the FPE fidelity scale.

The fidelity assessors also completed a survey seven months after the last fidelity assessment, answering questions on the feasibility of administering and scoring the fidelity scale. Questions addressed ease of finding information, making ratings, using various aspects of the scale, and the usefulness of different sources of information and the instructions.

### Data Analysis

After every fidelity review, we calculated the independent site-level fidelity scores for both fidelity assessors completing the review. The site-level fidelity score is defined as the sum of the item ratings divided by the number of items (that is, 13). To evaluate interrater reliability of the site fidelity ratings, we used the intraclass correlation coefficient (ICC) (McGraw and Wong 1996), based on a one-way random effects analysis of variance model for agreement between the two fidelity assessors on the FPE fidelity scale. We calculated a single ICC, based on 20 paired ratings for the five sites across four assessments.

We used consensus ratings in all subsequent analyses. To examine internal consistency of the FPE scale, we used Cronbach’s alpha, calculating an alpha coefficient for each time period. We examined the item distributions and site scores at 18 months, (mean, standard deviations, and distribution of scores) for full (rating = 5), adequate (= 4), and poor (= 1–3) fidelity.

Finally, we examined longitudinal patterns of fidelity both graphically and statistically. We examined sensitivity for change over time in fidelity using a one-way ANOVA repeated measures design with pairwise post hoc tests with Bonferroni correction for changes between baseline and each of the three follow-up assessments. We tabled frequency distributions of site fidelity scores over time, with specific attention to achievement of high fidelity ($\ge$ 4.0). Change over time was estimated by calculating the standardized mean difference effect size (Cohen’s d_z_) for within-subjects design (Lakens 2013). We examined feasibility using descriptive statistics and paired-sample t-tests for FPE item differences. All data analyses were done using SPSS version 25 (https://www.ibm.com/analytics/us/en/spss/spss-statistics-version/).

## Results

### Interrater Agreement

Over all items and time periods, exact agreement between assessors on items was good, averaging 88% (see, Table A in the Appendix). The mean exact agreement declined from 95% at baseline to 75–88% thereafter. High agreement at baseline may have been spurious due to many ratings of 1 reflecting a lack of FPE implementation. At the item level, mean agreement between assessors exceeded 80% on ten items and was under 80% on three items: 1 (*Family Intervention Coordinator*), 13 (*Stagewise Provision of Services*) and 14 (*Assertive Engagement and Outreach*).

### Interrater Reliability

Two fidelity assessors rated the FPE fidelity scales on four occasions at each of the 5 participating sites (100% completion rate). The intraclass correlation measuring interrater reliability (assuming two assessors) was excellent (0.98). For all subsequent analyses, we reported the findings based on consensus ratings.

### Internal Consistency

Internal consistency (Cronbach’s alpha) ranged from moderate to high: 0.96 (baseline), 0.79 (6 months), 0.97 (12 months), and 0.60 (18 months).

### Item Analysis

As shown in Table 1, the mean item scores for the five sites at 18 months ranged from 3.40 (Item 1: *Family Intervention Coordinator* and Item 14: *Assertive Engagement and Outreach* to 4.80 (Item 2: *Session Frequency*, and Item 8: *Coping Strategies*). Ratings significantly increased between baseline and 18 months on several items; *Long-term FPE, Psychoeducational Curriculum, Structured Problem Solving* and *Stage-wise Provision of Services*. Notably, by 18 months, ten of the items reached a mean score of 4.0 or above, which is the benchmark for adequate fidelity. Fidelity reviewers used the entire rating scale from 1 to 5 for all 13 items, suggesting that the rating scale captured the observed variability in actual practice.

**Table 1:** Item distributions for time change on the FAM fidelity scale 0–18 months (*N* = 5 sites)

	0 months	18 months	Difference 0 and 18 months	Distribution of fidelity ratings at 18 months		
Fidelity scale items	Mean (SD)	Mean (SD)	Significance p (paired t-test)	Poor 1–3	Adequate 4	Full 5
Family intervention coordinator	2.20 (1.64)	3.40 (1.67)	.178	3	0	2
Session frequency	3.40 (2.19)	4.80 (0.45)	.263	0	1	4
Long-term FPE	2.40 (1.34)	4.00 (1.41)	.003	2	0	3
Practitioner-consumer-family alliance	2.60 (1.82)	4.20 (0.84)	.099	1	2	2
Detailed family reaction	2.60 (2.19)	4.60 (0.55)	.129	0	2	3
Precipitating factors	2.60 (2.19)	4.60 (0.55)	.129	0	2	3
Coping strategies	2.60 (2.19)	4.80 (0.45)	.074	0	1	4
Psychoeducational curriculum	1.80 (1.79)	4.40 (0.89)	.025	1	1	3
Multimedia education	1.40 (0.89)	4.40 (1.41)	.099	4	0	1
Structured group sessions	2.40 (1.95)	4.00 (0.89)	.160	2	1	2
Structured problem solving	2.00 (1.73)	4.40 (0.89)	.042	1	1	3
Stage-wise provision of services	1.60 (1.34)	3.80 (1.64)	.040	1	2	2
Assertive engagement and outreach	3.00 (1.87)	3.40 (0.55)	.717	3	2	0

Items rated on a 5-point scale, with 5 = fully implemented

### Change over Time

**Fig. 1:**
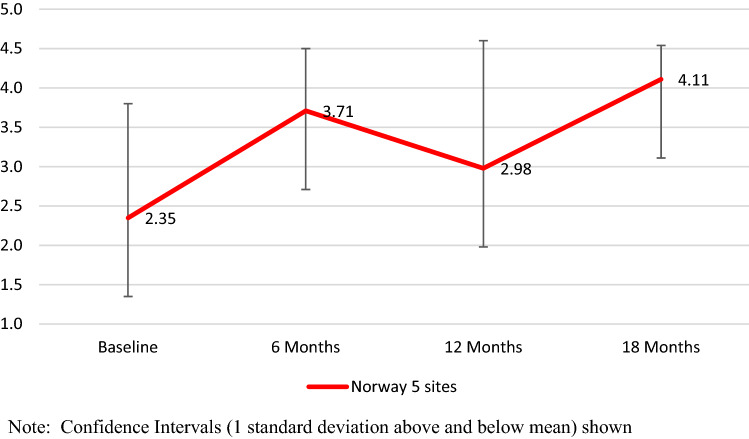
Mean family psychoeducation fidelity over time in full sample (five sites). Confidence intervals (one standard deviation above and below mean) shown

We visually inspected the graph of change across the 18-month period for the five sites, as shown in Fig. 1. At baseline, the mean site-level fidelity rating for the total scale was 2.35, suggesting that some implementation of family implementation was occurring at baseline, but nonetheless resulting in overall very low fidelity. By 6 months, mean fidelity had increased to 3.71, a mean increase of 1.35, but not significant, (t =  − 3.08, p = 0.22). At 12 months, the level of fidelity declined to 2.98, which was not significantly different from baseline (t =  − 0.53, p = 0.62) At 18 months, fidelity increased to 4.11, a mean increase of 1.75 from baseline, (t =  − 2.55, p = 0.38), thereby exceeding the benchmark for good fidelity. The standardized mean difference effect size (Cohen’s d_z_) was 1.14.

We also examined change over time looking at the percentage of sites attaining adequate fidelity (4.0 or higher) at each time period. At baseline, one site (20%) had already achieved adequate fidelity to FPE at baseline. By 12 months, one additional site had achieved adequate fidelity, and by 18 months, three sites (60%) achieved adequate fidelity.

### Feasibility

The 15 FPE fidelity assessors rated feasibility of the fidelity review process based on fidelity reviews both for sites receiving implementation support and seven control sites that did not. On average, fidelity assessors completed fidelity reviews at 11 sites across the four points in time. For most items assessors reported that both finding information and making ratings were easy. One exception was that, assessors found it difficult to find information on the quality of practitioner-consumer-family alliance. The interviews with clinicians were the most useful sources of information, while interviews with leaders and written procedures were less useful. They reported that the format of the fidelity scale was clearly set out and that the instructions were clear.

## Discussion

Overall the psychometric properties of the FPE fidelity scale were good. Fidelity assessors had acceptable levels of agreement on use of individual items in the FPE fidelity scale. The assessors in the study reached a high level of interrater reliability, indicating a very high degree of agreement. The fidelity scale also had good internal consistency at two of three follow-up assessments, suggesting that the 13 items comprising the FPE fidelity scale were measuring a unitary construct. Although not reaching statistical significance, the FPE scale increased substantially between baseline and 18 months suggesting that a longer follow up period might be useful in order to investigate this. The whole rating range (from 1 to 5) was used for most items.

In this study five sites improved in FPE fidelity over an 18-month period. By 18 months follow up the sites reached a mean fidelity by 4.0, the benchmark for good fidelity. The mean level of fidelity for this was comparable to two prior US implementation studies (Kealey et al. 2015). Based on six fidelity reviews, fidelity assessors indicated that assessing FPE fidelity was feasible; interviewing clinicians was the most useful source of information for making fidelity ratings.

The FPE fidelity scale was judged to be equally suited to evaluate both the multi-family (one site) as well as the single-family format. Most sites considered the single-family approach more feasible. We conclude that the FPE scale is feasible for evaluating clinical services when structured family interventions are offered. The scale may be used in other populations after making appropriate adaptations to the fidelity scale, as a common approach used by fidelity scale developers (Bond and Drake 2019).

This study highlights several feasibility and efficiency challenges to consider when introducing fidelity measurement in clinical practice. With adequate resources, an evidence-based intervention like FPE can be implemented with acceptable fidelity in ordinary mental healthcare units. In implementing FPE-systems in ordinary clinical practice, a system of regularly monitoring could be useful (Bond et al. 2009). Unfortunately, even if there is a strong evidence base and support among clinicians for offering FPE to persons with severe mental illness, there is still a gap between such support and implementation into “real world” clinical settings.

### Strengths and Limitations

The strength of the study was implementation in a national, public-funded, “real world” clinical system serving nearly all patients with psychotic disorders in each health trust’s catchment area. Limitations included the small number of study sites for generalizability, the therapist self-report bias and the lack of interviews with patients and their families, and the absence of direct observation of FPE sessions which affects the FPE fidelity scale validity.

### Conclusion and Implications

The current study is one of few (Kealey et al. 2015; McHugo et al. 2007) investigating both use and psychometric properties of the FPE fidelity scale. The FPE fidelity scale has good psychometric properties and feasibility for evaluating the implementation of FPE programs. We conclude that the FPE scale is feasible for evaluating clinical services where structured family interventions are offered. In future revisions of the FPE fidelity scale collection of observational fidelity data should be considered. Nevertheless, a larger study could provide more robust conclusions and investigate the predictive validity of the FPE fidelity scale on long-term outcome.

## Electronic Supplementary Material

Below is the link to the electronic supplementary material.

Supplementary file 1 (DOCX 14 kb)

Supplementary file 2 (DOC 64 kb)
