# Supplementary material for: The Family Psychoeducation Fidelity Scale: Psychometric Properties
Source: Adm Policy Ment Health. 2020 Apr 23;47(6):894–900. doi: 10.1007/s10488-020-01040-3 (PMC7547979; doi:10.1007/s10488-020-01040-3)
Supplement: Supplementary file 1 — (DOCX 14 kb) [file 10488_2020_1040_MOESM1_ESM.docx]

**Online appendix**

**Table A. Percentage Agreement Between Fidelity Assessors on Individual Items (5 units)**

| **Item** | **Description** | **Baseline%** | **6 Months%** | **12 Months%** | **18 Months%** | **Mean%** |
| --- | --- | --- | --- | --- | --- | --- |
| 1 | **Family intervention coordinator** | 80 | 20 | 100 | 80 | 70 |
| 2 | **Session frequency** | 80 | 100 | 100 | 80 | 90 |
| 3 | **Long-term FPE** | 80 | 80 | 100 | 100 | 90 |
| 4 | **Quality of practitioner-consumer-family alliance** | 100 | 80 | 80 | 60 | 80 |
| 5 | **Detailed family reaction** | 100 | 100 | 100 | 80 | 95 |
| 6 | **Precipitating factors** | 100 | 100 | 100 | 80 | 95 |
| 8 | **Coping strategies** | 100 | 100 | 80 | 80 | 90 |
| 9 | **Psychoeducational curriculum** | 100 | 80 | 80 | 60 | 80 |
| 10 | **Multimedia education** | 100 | 60 | 80 | 80 | 80 |
| 11 | **Structured group sessions** | 100 | 80 | 60 | 80 | 80 |
| 12 | **Structured problem solving** | 100 | 100 | 60 | 80 | 80 |
| 13 | **Stage-wise provision of services** | 100 | 60 | 60 | 80 | 75 |
| 14 | **Assertive engagement and outreach** | 100 | 80 | 80 | 40 | 75 |
|  | **Mean Agreement for 13 Items** | **95.38** | **87.69** | **83.08** | **75.38** | **87.88** |
