# Supplementary material for: The Family Psychoeducation Fidelity Scale: Psychometric Properties
Source: Adm Policy Ment Health. 2020 Apr 23;47(6):894–900. doi: 10.1007/s10488-020-01040-3 (PMC7547979; doi:10.1007/s10488-020-01040-3)
Supplement: Supplementary file 2 — (DOC 64 kb) [file 10488_2020_1040_MOESM2_ESM.doc]

|  | | **1** | | **2** | **3** | | **4** | | **5** | |
| --- | --- | --- | --- | --- | --- | --- | --- | --- | --- | --- |
| **1. Family Intervention Coordinator.** One clinical administrator is designated as overseer of the FPE program for a substantial portion of his/her job (time depends on size of program). This person's activities include:   - Establish, monitor, and automate family intake & engagement procedures - Assign potential FPE clients to FPE staff and monitor/adjust caseloads - Arrange for training of new staff - Arrange for continuing education of existing staff - Arrange supervision for staff | | Agency does not have a designated position | | Agency has a designated position who performs 1 or 2 of the tasks | Agency has a designated position who performs  3 of the tasks | | Agency has a designated position who performs  4 of the tasks | | Agency has a designated position who performs all tasks | |
| 2. Session Frequency for FPE. | | < Every 3 months | | Every 3 months | Every 2 months | | Monthly | | At least twice a month | |
| **3. Long-Term FPE.** | | Most families receive less than 6 months of FPE sessions | | Most families receive 6-7 months of FPE sessions | Most families receive between 7-8 months of FPE sessions | | Most families receive 8-9 months of FPE sessions | | >90% families receive at least 9 months of FPE sessions | |
| **4. Quality of Clinician-Family Alliance.** In individual or group sessions, the clinician engages family members and consumer with warmth, empathy, acceptance and attention to each individual’s needs and desires. | | High dropout rate | | Sources indicate that clinician-family alliance often poor leading to high drop-out | Sources indicate alliance is inconsistent or barely adequate leading to moderate drop-out, OR information is inconsistent | | Sources indicate a fairly strong clinician-family alliance | | Sources consistently  indicate a strong clinician-  family alliance | |
| **5. Detailed Family Reaction.** In individual or single-family ***joining*** sessions, the clinician(s) help to identify and specify the family's reaction to their relative's mental illness. | | There is consistent evidence for <33% of involved families | | There is consistent evidence for 33-49% of involved families | There is consistent evidence for 50%-64% of involved families | | There is consistent evidence for 65%-79% of involved families | | There is consistent evidence (documentation in the chart and reports by clinicians, consumers, & families) for 80% or more of involved families | |
|  | **1** | | **2** | | | **3** | | **4** | | **5** |
| **6. Precipitating Factors.** In individual or single-family ***joining*** sessions, the clinician(s) help to identify and specify precipitating factors to their relative's mental illness. | There is consistent evidence for <33% of involved families | | There is consistent evidence for 33-49% of involved families | | | There is consistent evidence for 50%-64% of involved families | | There is consistent evidence for 65%-79% of involved families | | There is consistent evidence (documentation in the chart and reports by clinicians, consumers, & families) for 80% or more of involved families |
| **7. Prodromal Signs.** In individual or single-family ***joining*** sessions, the clinician(s) help to identify and specify prodromal signs and symptoms of their relative's mental illness. | There is consistent evidence for <33% of involved families | | There is consistent evidence for 33-49% of involved families | | | There is consistent evidence for 50%-64% of involved families | | There is consistent evidence for 65%-79% of involved families | | There is consistent evidence (documentation in the chart and reports by clinicians, consumers, & families) for 80% or more of involved families |
| **8. Coping Strategies.** In individual or single-family ***joining*** sessions, the clinician(s) help to identify, describe, and clarify coping strategies used by families and teach effective coping strategies. | There is consistent evidence for <33% of involved families | | There is consistent evidence for 33-49% of involved families | | | There is consistent evidence for 50%-64% of involved families | | There is consistent evidence for 65%-79% of involved families | | There is consistent evidence (documentation in the chart and reports by clinicians, consumers, & families) for 80% or more of involved families |
| **9. Educational Curriculum.** FPE uses a standardized curriculum to teach families about mental illness. The curriculum covers six topics:   - Psychobiology - Diagnosis - Treatment & rehabilitation - Reactions to experiencing psychosis   as a family   - Relapse prevention - Family guidelines | <33% of involved families receive a standardized educational curriculum covering all 6 topics, there is no standardization, OR only 1-2 topics are covered | | 33% - 49% of involved families receive a standardized educational curriculum covering all 6 topics OR more families receive 3 topics | | | 50% - 64% of involved families receive a standardized educational curriculum covering all 6 topics OR more families receive 4-5 topics | | 65%-79% of involved families receive a standardized educational curriculum covering all 6 topics | | 80% or more of involved families receive a standardized educational curriculum covering all 6 topics |
| **10. Multimedia Education.** Educational materials on illness, treatment, and guidelines are provided with choices in several formats (e.g., written, video, websites). | No educational materials available | | Only 1 format available and it is barely mentioned and the majority of families are unaware of its existence | | | Only 1 format easily accessible. The other two formats either not available or access is very limited | | Only 2 of 3 formats easily accessible The third format is not available or is inaccessible (e.g., no copies available) | | All 3 formats (written, video, & websites) are available and made accessible to family members repeatedly |

|  | **1** | **2** | **3** | **4** | **5** |
| --- | --- | --- | --- | --- | --- |
| **11. Structured Group Sessions.** Multiple family groups follow a structured procedure that includes:   - Beginning socialization - Follow-up of previous week’s   problem(s)   - Go-round - Response to each family - Problem solving - End with socialization | No more than 2 of 6 components of structured group sessions are represented | 3 of 6 components of structured group sessions are represented | 4 of 6 components of structured group sessions are represented | 5 of 6 components of structured group sessions are represented | All 6 components of structured group sessions are represented |
| **12. Structured Problem-Solving** **Technique.** In individual or group sessions, the clinician(s) use a standardized approach:   - Identify a problem for one   consumer/family   - Define the problem - Generate 8 or more solutions - Review pros & cons - Select a solution - Develop specific & individualized   tasks & plans | No more than 2 of 6 components of the structured problem-solving technique are utilized | 3 of 6 components of the structured problem-solving technique are utilized | 4 of 6 components of the structured problem-solving technique are utilized | 5 of 6 components of the structured problem-solving technique are utilized | All 6 components of the structured problem-solving technique are utilized |
| **13. Stage-wise Provision of Services.** FPE services are provided in the following order:   1. Engagement 2. 3-5 ***joining*** sessions covering:    - Detailed family reaction    - Precipitating factors    - Prodromal signs    - Identification & teaching of   coping skills   1. 3) Educational workshop 2. 4) Multi-family group | Families are entered into the multi-family group with minimal or no engagement, no joining sessions, or no education | Engagement is minimal and **only** **one** *joining* session is completed prior to the families’ entry into the multi-family group. Education is delayed or absent | Engagement and **two** *joining* sessions are completed prior to the families’ entry into the multi-family group. Education is delayed or absent | Most steps are done in order; however, the families are brought into the multi-family group before education is provided or before three *joining* sessions are completed | Engagement, all **three** *joining* sessions, and education are completed in the order prior to the families’ entry into the multi-family group |

|  | **1** | **2** | **3** | **4** | **5** |
| --- | --- | --- | --- | --- | --- |
| 14. Assertive Engagement & Outreach. FPE clinicians actively engage all potential consumers and family members via phone, mail, or in person (in the clinic or in the community) on an on-going basis to educate them about FPE and give them the opportunity to make an informed decision about participation. | FPE clinicians do not assertively engage potential consumers and family members | FPE clinicians attempt to engage potential consumers and family members only once by phone or mail as a part of initial engagement | FPE clinicians attempt to engage some potential consumers and family members only 1-2 times via all necessary contact means as a part of initial engagement | FPE clinicians actively engage some potential consumers and family members via all necessary contact means on a time-limited basis as a part of initial engagement | FPE clinicians actively engage all potential consumers and family members via all necessary contact means as a part of engagement on an on-going basis. FPE practitioners demonstrate tolerance of different levels of readiness using gentle encouragement |

Web site of the Center for Mental Health Services of the Substance Abuse and Mental Health Services Administration
(mentalhealth.samhsa.gov/cmhs/communitysupport/toolkits)
